# Variable Penetrance and Expressivity of a Rare Pore Loss-of-Function Mutation (p.L889V) of Nav1.5 Channels in Three Spanish Families

**DOI:** 10.3390/ijms25094686

**Published:** 2024-04-25

**Authors:** María Gallego-Delgado, Anabel Cámara-Checa, Marcos Rubio-Alarcón, David Heredero-Jung, Laura de la Fuente-Blanco, Josu Rapún, Beatriz Plata-Izquierdo, Sara Pérez-Martín, Jorge Cebrián, Lucía Moreno de Redrojo, Belén García-Berrocal, Eva Delpón, Pedro L. Sánchez, Eduardo Villacorta, Ricardo Caballero

**Affiliations:** 1 Department of Cardiology, CSUR Cardiopatías Familiares, Institute of Biomedical Research of Salamanca (IBSAL), Complejo Asistencial Universitario de Salamanca, Gerencia Regional de Salud de Castilla y León (SACYL), 37007 Salamaca, Spain; m.gallego.delgado@gmail.com (M.G.-D.);; 2 Centro de Investigación Biomédica en Red en Enfermedades Cardiovasculares (CIBERCV), 28029 Madrid, Spainmr956@cam.ac.uk (M.R.-A.); jrapun01@ucm.es (J.R.); jorgeceb@ucm.es (J.C.); rcaballero@ucm.es (R.C.); 3 Department of Pharmacology, School of Medicine, Instituto de Investigación Sanitaria Gregorio Marañón, Universidad Complutense, 28040 Madrid, Spain; 4 Department of Biochemistry, CSUR Cardiopatías Familiares, Institute of Biomedical Research of Salamanca (IBSAL), Complejo Asistencial Universitario de Salamanca, Gerencia Regional de Salud de Castilla y León (SACYL), 37007 Salamaca, Spain; 5 Department of Pediatrics, Institute of Biomedical Research of Salamanca (IBSAL), Complejo Asistencial Universitario de Salamanca, Gerencia Regional de Salud de Castilla y Leon (SACYL), CIBERCV, 37007 Salamaca, Spain; bplata@usal.es

**Keywords:** SCN5A, Nav1.5, mutation, Brugada syndrome, cardiac conduction defect, dilated cardiomyopathy, phenotypic penetrance, phenotypic variability

## Abstract

A novel rare mutation in the pore region of Nav1.5 channels (p.L889V) has been found in three unrelated Spanish families that produces quite diverse phenotypic manifestations (Brugada syndrome, conduction disease, dilated cardiomyopathy, sinus node dysfunction, etc.) with variable penetrance among families. We clinically characterized the carriers and recorded the Na^+^ current (I_Na_) generated by p.L889V and native (WT) Nav1.5 channels, alone or in combination, to obtain further insight into the genotypic–phenotypic relationships in patients carrying *SCN5A* mutations and in the molecular determinants of the Nav1.5 channel function. The variant produced a strong dominant negative effect (DNE) since the peak I_Na_ generated by p.L889V channels expressed in Chinese hamster ovary cells, either alone (−69.4 ± 9.0 pA/pF) or in combination with WT (−62.2 ± 14.6 pA/pF), was significantly (n ≥ 17, *p* < 0.05) reduced compared to that generated by WT channels alone (−199.1 ± 44.1 pA/pF). The mutation shifted the voltage dependence of channel activation and inactivation to depolarized potentials, did not modify the density of the late component of I_Na_, slightly decreased the peak window current, accelerated the recovery from fast and slow inactivation, and slowed the induction kinetics of slow inactivation, decreasing the fraction of channels entering this inactivated state. The membrane expression of p.L889V channels was low, and in silico molecular experiments demonstrated profound alterations in the disposition of the pore region of the mutated channels. Despite the mutation producing a marked DNE and reduction in the I_Na_ and being located in a critical domain of the channel, its penetrance and expressivity are quite variable among the carriers. Our results reinforce the argument that the incomplete penetrance and phenotypic variability of *SCN5A* loss-of-function mutations are the result of a combination of multiple factors, making it difficult to predict their expressivity in the carriers despite the combination of clinical, genetic, and functional studies.

## 1. Introduction

The *SCN5A* gene encodes the α-subunit of the voltage-gated sodium channel, known as Nav1.5. This protein generates the sodium current (I_Na_), which is responsible for the upstroke (phase 0) of cardiac action potentials (AP). I_Na_ critically determines the excitability and conduction of electrical impulses in the heart, predominantly in the atrioventricular (AV) bundle, His and Purkinje fibers, and atrial and ventricular myocardium [1].

*SCN5A* variants cause a wide spectrum of arrhythmogenic hereditary diseases associated with sudden cardiac death (SCD). Overall, *SCN5A* mutations can cause loss- (LOF) or gain-of-function (GOF) of Nav1.5 channels or a combination of both [2,3]. LOF mutations are commonly the consequence of defects in channel trafficking, expression, or biophysical properties. Phenotypes associated with LOF variants include Brugada syndrome (BrS), progressive cardiac conduction defect (PCCD), and sinus node dysfunction (SND), among others [4]. GOF mutations produce an increased Na^+^ influx (I_NaL_) during the plateau phase of the AP as a consequence of the disruption of the channel’s fast inactivation, slowed inactivation course, and/or increased window current and are associated with type 3 long QT syndrome. Interestingly, a single mutation can lead to multiple phenotypes in different carriers and even produce simultaneous LOF and GOF alterations on the Nav1.5 channels (overlap syndrome) [5]. Furthermore, some *SCN5A* mutations also lead to structural cardiomyopathies such as dilated cardiomyopathy (DCM) [2,6]. It is well established that due to incomplete penetrance, delayed and variable expression, and marked phenotypic heterogeneity, the interpretation of novel, rare *SCN5A* variants is extremely complicated.

Here, we have functionally analyzed the novel p.L889V variant of Nav1.5 channels found in three unrelated Spanish families. We have tried to weigh the influence of the mutation-induced I_Na_ alterations as well as those factors that are supposed to determine the variable penetrance and expressivity since we have the opportunity to compare the phenotypes of the three probands and their respective relatives.

## 2. Results

Between 2013 and 2022, *SCN5A* was sequenced as part of the genetic analysis of 452 consecutive probands (72% male; age at diagnosis 50 ± 18 years; 5% pediatric cases, younger than 15 years) evaluated in the unit for inherited cardiovascular diseases of the Hospital Universitario de Salamanca (Spain), resulting in 11 actionable *SCN5A* variants. Interestingly, among them, we identified in three apparently unrelated probands the heterozygous g.3.38627304G > C (NM_198056.2:c.2665C > G) variant, which was classified as pathogenic by several predictive algorithms (Table S1) and encodes p.L889V Nav1.5 channels. The mutated residue is highly conserved among different species and lays in the first helical portion of the P loop between the S5 and S6 segments of DII (Figure S1). The bottom part of Figure S1A shows the alignment of the amino acids of the first helix of the P loop regions within the four domains of Nav1.5. Interestingly, the only fully conserved residue among the four domains is the leucine residue equivalent to L889, suggesting the importance of the existence of a ring of four leucine residues at this level for channel function. The p.L889V variant has not been annotated so far in public repositories such as gnomAD or ClinVar (https://gnomad.broadinstitute.org/ and https://www.ncbi.nlm.nih.gov/clinvar/, accessed on 4 April 2024). Thus, according to the guidelines proposed by the American College of Medical Genetics and Genomics (ACMG) [7], the mutation meets two criteria supporting its moderate pathogenicity (it is located in a critical functional domain (PM1) and appears at extremely low frequency in the population (PM2)). Moreover, computational evidence supports its deleterious effects (supporting evidence of pathogenicity (PP3)).

### 2.1. Clinical Description of Probands and Their Families

**Figure 1:**
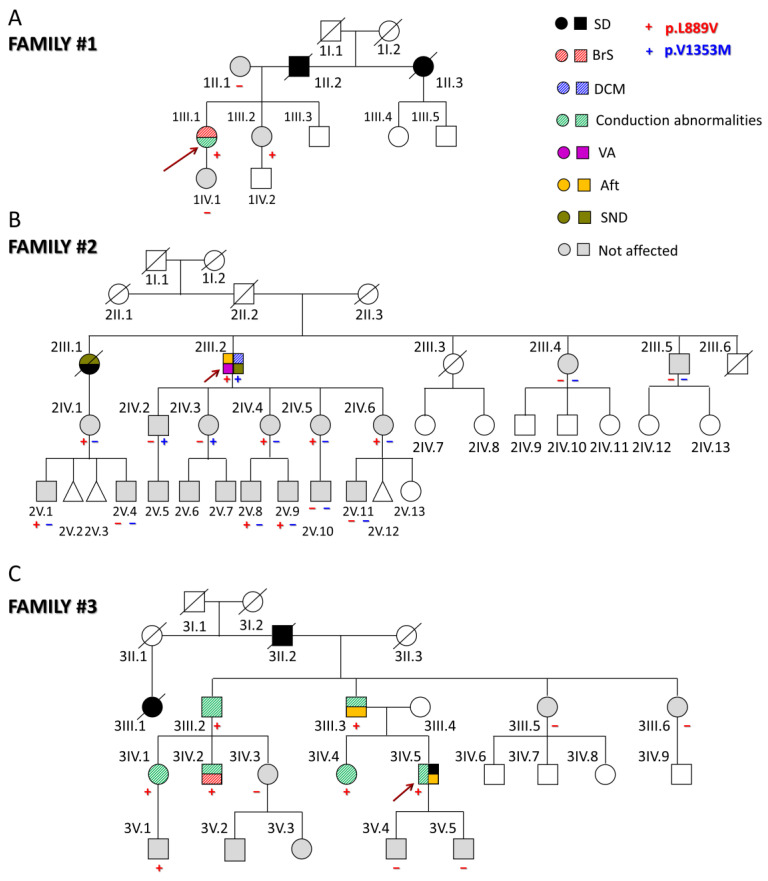
(**A**–**C**) Pedigrees of the three families analyzed in this study. The arrows indicate the probands. Circles, squares, and triangles represent females, males, and abortions, respectively. Deceased individuals are marked with a diagonal line. White symbols represent subjects that were not studied; the correspondence of other symbols/colors is indicated in the figure. Aft: atrial flutter; BrS: Brugada syndrome; DCM: dilated cardiomyopathy; SD: sudden death; SND: sinus node dysfunction; VA: ventricular arrhythmias.

The first proband (1III.1) was a 31-year-old woman (Figure 1A) who presented with episodes of paroxysmal tachycardia, predominantly nocturnal. The ECG showed sinus rhythm with a Brugada type I pattern (Figure S2). Later, she developed a nocturnal grade 2 type I AV block, and a subcutaneous Holter ECG was implanted due to the worsening of palpitations. However, she had no echocardiographic abnormalities and had not experienced syncope so far. Her father (1II.2) and paternal aunt (1II.3) suddenly died when they were 44 (because of myocardial infarction as demonstrated by the autopsy) and 54 years old, respectively. None of the first-degree relatives clinically studied (1II.1, 1III.2, and 1IV.1) suffer from any arrhythmia type, and only her sister (1III.2), but not her mother and daughter, carries the p.L889V Nav1.5 variant.

The second proband (2III.2) experienced sustained monomorphic ventricular tachycardia that was poorly tolerated and was cardioverted when he was 64 (Figure 1B). Thereafter, he developed atrial flutter (Aft) and SND, so a cavotricuspid isthmus ablation and implantation of a dual-chamber defibrillator were performed (Figure S2). At that time, mild systolic dysfunction was evident (left ventricular ejection fraction (LVEF) 45%). During follow-up, despite optimized medical treatment, the LVEF dropped to 36% with clinical worsening to NYHA III; therefore, an upgrade to cardiac resynchronization therapy was indicated. After this, LVEF and functional class recovered and have been maintained up until today. The genetic analysis demonstrated that this proband also carries the heterozygous g.3.38560335C > T *SCN5A* variant (NM_198056.2:c.4057G > A) encoding p.V1353M Nav1.5 channels. This variant was already annotated (rs199473233) with an allele frequency of 0.001487% and related to BrS [8,9] although there are conflicting data regarding its pathogenicity since in ClinVar, nine submitters classified it as a variant of unknown significance and one as benign.

The proband’s parents had apparently died of non-cardiac causes, and a paternal aunt (2II.1) had a history of heart failure (without reliable medical records) (Figure 1B). The proband’s older sister (2III.1) had died suddenly at the age of 26 during the puerperium (pulmonary embolism was suspected). A year earlier, she had a pacemaker implanted due to SND (tachycardia–bradycardia syndrome). Two other younger siblings (2III.3 and 2III.6) had died of non-cardiac causes, while his siblings, 2III.4 and 2III.5, had a normal ECG, Holter, and echocardiograms. The proband’s niece (2IV.1) (daughter of 2III.1) carries the p.L889V variant but does not have any arrhythmic or structural cardiomyopathy. She has two sons (2V.1 and 2V.4), one of whom (2V.1) also carries the p.L889V mutation. Among the five proband’s children, the three younger daughters (2IV.4, 2IV.5, and 2IV.6) carry the p.L889V mutation, while 2IV.2 and 2IV.3 carry the p.V1353M variant. These results demonstrate that each allele of the proband encoded one of the variants separately (compound heterozygosity). Among the eight proband’s grandchildren, only four were genotyped, and 2V.8 and 2V.9 also carry the p.L889V variant. Interestingly, in this family, there are eight and three alive carriers of the p.L889V and p.V1353M mutations, respectively, and except for the proband, none of them have any clinical manifestations.

The third proband (3IV.5) suffered a cardiac arrest due to ventricular fibrillation at the age of 46 (Figure 1C). Previously, he had experienced syncopes since he was 17 years old, and his ECG documented a first-degree AV block (Figure S2). Later, he suffered another syncope coincident with Aft that was ablated. At the age of 28, an infra-Hisian block was documented, and a pacemaker was implanted after a new syncopal episode. Finally, he received an implantable cardioverter device. Imaging tests have not shown any structural heart disease. His father (3III.3) had a similar medical history, with pacemaker implantation at an early age due to syncope and conduction disorders and later episodes of Aft and atrial fibrillation (AF). The proband’s grandfather (3II.2) and a cousin of the proband’s father (3III.1) suddenly died when they were 53 and 26, respectively. The proband’s paternal uncle (3III.2) required pacemaker implantation and carries the p.L889V variant. Two cousins (3IV.1 and 3IV.2), descendants of 3III.2, are also carriers and have conduction alterations in the ECG. Furthermore, 3IV.2 exhibits a BrS type I pattern. The proband’s sister (3IV.4) is also positively genotyped and exhibits conduction abnormalities on the ECG. None of the proband’s sons (3V.4 and 3V.5) carry the variant, but a nephew (3V.1) does, and he currently presents a right bundle branch block. Therefore, in this family, six of the seven carriers had intraventricular conduction abnormalities.

Overall, thirty-three relatives from the three families have been evaluated, identifying five new cases with a positive genotype, nine asymptomatic carriers, and nineteen non-carriers of the p.L889V variant. The adult carriers (older than 16 years old) of the p.L889V variant had longer PR (with a higher prevalence of first-degree AV block) and lower age when first arrhythmia (Table S2) than no carriers. The data further support the evidence of the pathogenicity of the variant since it was associated with different types of arrhythmias and cardiac diseases in the three families (PP1 according to the ACMG guidelines). Next, we decided to record the I_Na_ generated by p.L889V Nav1.5 channels.

### 2.2. Functional Analysis of p.L889V Nav1.5 Channels

**Figure 2:**
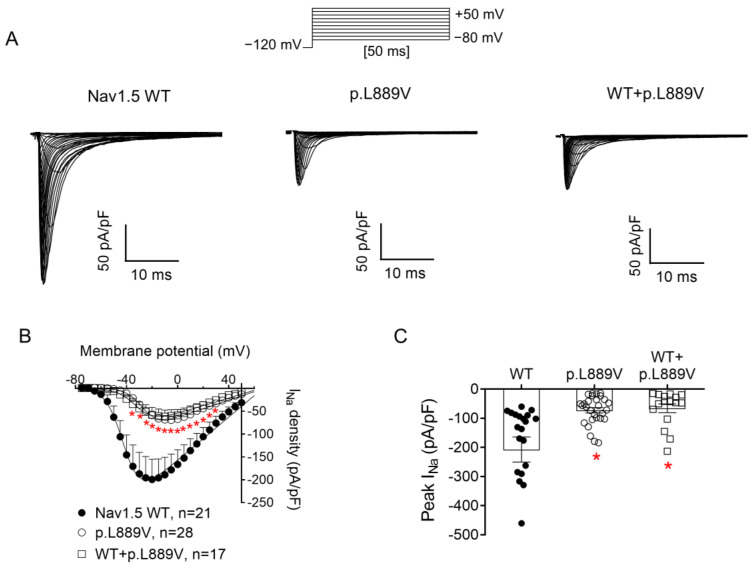
(**A**) I_Nav1.5_ traces recorded by applying the protocol shown at the top in three different CHO cells transfected or not with the cDNA encoding WT, p.L889V, or WT and p.L889V Nav1.5. (**B**,**C**) Mean current density–voltage (**B**) and peak current density values (**C**) for I_Nav1.5_ recorded in the three experimental groups. In (**B**,**C**), each point/bar represents the mean ± SEM of ‘n’ experiments/cells and in (**C**), each dot represents one single experiment. In (**B**,**C**), * *p* < 0.05 vs. WT. ANOVA followed by Tukey’s test and a multilevel mixed-effects model.

**Figure 3:**
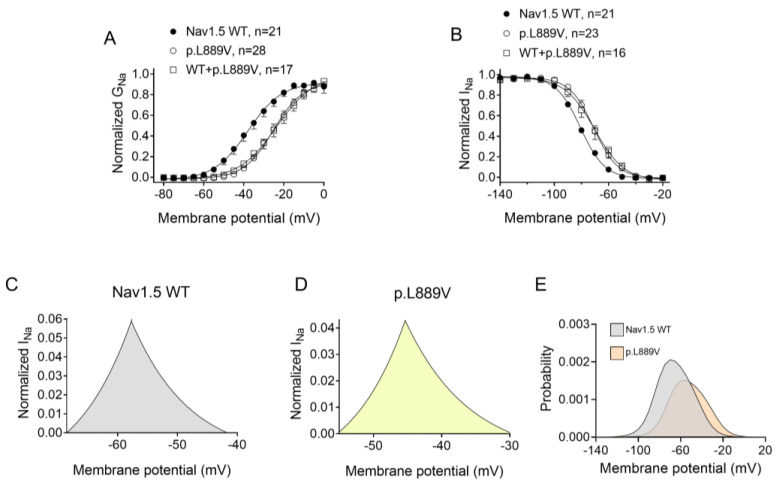
(**A**,**B**) Normalized conductance–voltage (activation) (**A**) and inactivation (**B**) curves for I_Nav1.5_ recorded in CHO cells transfected or not with the cDNA encoding WT, p.L889V, or WT and p.L889V Nav1.5. The solid lines represent the fit of a Boltzmann function to the data. Each point represents the mean ± SEM of the number of experiments indicated in the figures. ANOVA followed by Tukey’s test and multilevel mixed-effects model. (**C**–**E**) Expanded representation of the overlapping area between the activation and inactivation curves (window current) for I_Nav1.5_ recorded in CHO expressing WT (**C**) or p.L889V (**D**) Nav1.5 and the probability of being within this window (**E**).

Figure 2A shows I_Nav1.5_ traces recorded in three different Chinese hamster ovary (CHO) cells transiently transfected with the cDNAs encoding human WT, p.L889V, or WT and p.L889V Nav1.5 channels by applying the pulse protocol shown at the top, while Figure 2B shows the I_Nav1.5_ density–voltage curves. Cells were always co-transfected with the cDNA encoding Navβ1 proteins. The maximum I_Nav1.5_ density generated by p.L889V (−69.4 ± 9.0 pA/pF, n = 28) was significantly lower than that generated by WT Nav1.5 channels (−199.1 ± 44.1 pA/pF, n = 21) (Figure 2A–C). Furthermore, the current generated by the variant peaked at more positive potentials (−5 mV vs. −20 mV) (Figure 2B). Considering the heterozygous condition of mutant carriers, we co-transfected cells with WT and p.L889V Nav1.5 in a 0.5:0.5 ratio. Interestingly, the density of the I_Nav1.5_ generated by the co-transfection was similar (−62.2 ± 14.6 pA/pF, n = 17) to that generated in the presence of the mutant alone (*p* > 0.05) and significantly smaller than that generated by WT (*p* < 0.05) (Figure 2A–C). These results strongly suggest that the variant produces a marked dominant negative effect (DNE). To determine the consequences of the variant on the voltage dependence of activation, conductance–voltage relationships were constructed from the current density–voltage data (see Supplementary Materials). p.L889V channels, either alone or in combination with WT channels, were activated at more positive potentials compared to WT (Figure 3A), an effect that would contribute to the variant-induced reduction in the current density. In fact, the midpoint of the curve (Vh) was shifted from −38.5 ± 2.6 to −26.5 ± 1.6 mV (*p* < 0.05), without modifications in the slope (*p* > 0.05). To identify possible effects on the voltage dependence of channel availability, we constructed the steady-state inactivation curves using a standard double-pulse protocol. p.L889V Nav1.5 shifted the Vh of the inactivation curves from −80.8 ± 2.3 to −70.2 ± 2.3 mV (n ≥ 21, *p* < 0.05) without modifying the slope (*p* > 0.05) (Figure 3B). Strikingly, the voltage dependence of the activation and inactivation of WT and p.L889V channels was almost identical to that of the p.L889V variant alone (n ≥ 17, *p* > 0.05) (Figure 3A,B).

**Figure 4:**
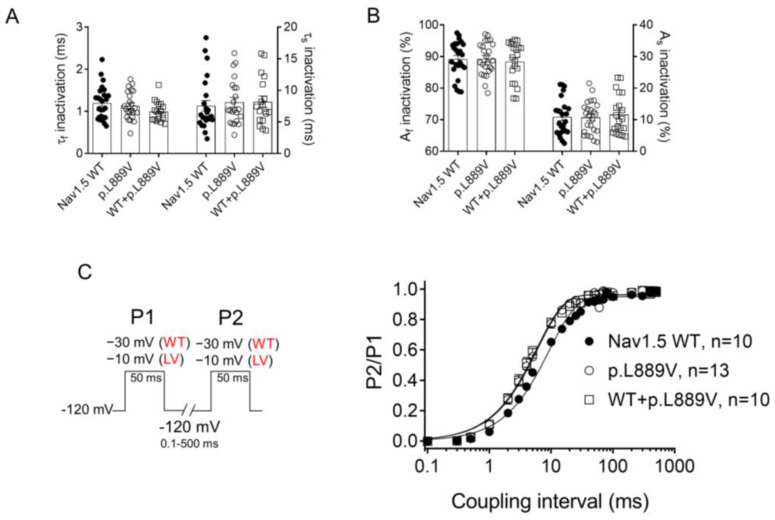
(**A**,**B**) Fast and slow time constants (**A**) and relative amplitudes (**B**) of the exponential components obtained by fitting a biexponential function to the decay of the currents recorded in CHO cells transfected or not with the cDNA encoding WT (at −30 mV), p.L889V (at −10 mV), or WT and p.L889V (at −10 mV) Nav1.5. (**C**) Time course of reactivation for I_Nav1.5_ recorded using the protocol shown on the left in CHO cells transfected or not with the cDNA encoding WT, p.L889V, or WT and p.L889V Nav1.5. The solid lines represent the fit of a monoexponential function to the data. WT and LV: membrane potential when recording I_Na_ generated by WT and p.L889V channels, respectively. In (**A**–**C**), bars/points represent the mean ± SEM of ‘n’ experiments/cells and in A-B, each dot represents one single experiment. ANOVA followed by Tukey’s test and multilevel mixed-effects model.

**Figure 5:**
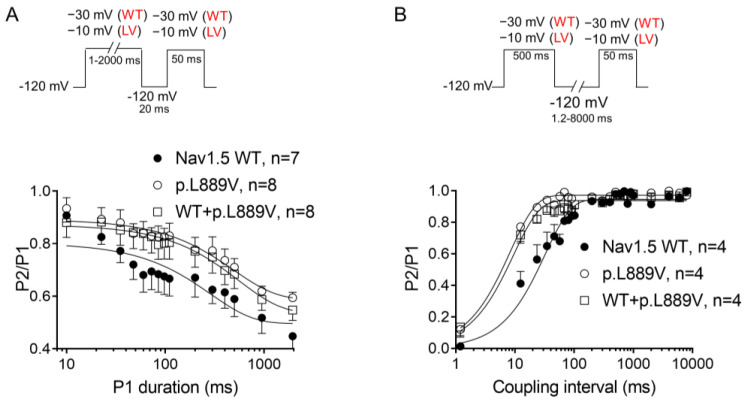
(**A**,**B**) Time course of development (**A**) and recovery (**B**) of/from slow inactivation for currents recorded in CHO cells transfected with the cDNA encoding WT, p.L889V, or WT and p.L889V Nav1.5 assessed with the double-pulse protocols shown at the top of each panel. WT and LV: membrane potential when recording I_Na_ generated by WT and p.L889V channels, respectively. In (**A**,**B**), the solid lines represent the fit of a monoexponential function to the data, and each point represents the mean ± SEM of the number of experiments indicated in the figures. ANOVA followed by Tukey’s test and multilevel mixed-effects model.

The overlap of the activation and steady-state inactivation of Na^+^ channels identifies a range of voltages (i.e., window), where the channels have a small probability of being partially but not fully inactivated (Figure 3C,D). We also calculated the probability of being within the window from the product of the fitted activation and steady-state inactivation parameters (Figure 3E). Compared with the window current generated by native channels, that of mutated channels is smaller and peaks at more depolarized potentials (from 0.21% at −70 mV for WT channels to 0.15% at −55 mV for p.L889V channels). The effects of the combination, which were very similar to those produced by the variant alone, were omitted from Figure 3C–E for clarity. Figure 4A,B depicts the fast (τ_f_) and slow (τ_s_) time constants and the amplitude of the respective components yielded by the biexponential fit to the decay of the peak I_Nav1.5_ traces in cells expressing WT, p.L889V, or WT and p.L889V. Neither the presence of the variant alone nor in combination with WT channels modified the kinetics of fast inactivation (*p* < 0.05). Recovery from fast inactivation was analyzed by applying two test pulses of identical duration (50 ms) with increasing coupling intervals (from 0.1 to 500 ms) (Figure 4C). The voltage of the test pulses was different for WT (−30 mV) and for p.L889V and WT and p.L889V channels (−10 mV), considering the p.L889V voltage-dependent effects. In any case, the holding and the potential between pulses were −120 mV. Recovery time course data were fitted by monoexponential functions yielding time constants (τ_re_) of 10.6 ± 1.9 (n = 10), 5.8 ± 0.7 (n = 13), and 5.4 ± 0.9 ms (n = 10) for WT, p.L889V, and WT and p.L889V Nav1.5 channels, respectively. Therefore, recovery from fast inactivation of p.L889V either alone or in combination with WT channels was significantly (*p* < 0.05) faster than that of WT channels. Nav1.5 channel inactivation accumulates when depolarization persists in a range of hundreds of milliseconds (slow or intermediate inactivation) [10]. Therefore, we next analyzed the effects of the variant on the development of slow inactivation by applying a pre-pulse to −30 (WT) or −10 mV (p.L889V and WT and p.L889V) of progressively increasing duration (1–2000 ms) followed by a test pulse to −30 (WT) or −10 mV (p.L889V and WT and p.L889V). Figure 5A shows that transfection with p.L889V slowed the induction kinetics (from τ = 296 ± 142 to 526 ± 168 ms, *p* < 0.05) and decreased the fraction of channels entering the slow inactivated state (from 0.55 ± 0.06 to 0.40 ± 0.02, n ≥ 7, *p* < 0.05). Possible effects on the recovery from the slow inactivation were determined by applying a 500 ms pulse to −30 (WT) or −10 mV (p.L889V and WT and p.L889V), followed by a pulse to −120 mV of progressively increasing duration (1.2–8000 ms), and by a 50 ms pulse to −30 (WT) or −10 mV (p.L889V and WT and p.L889V). The variant significantly accelerated recovery from slow inactivation since it decreased the time constant of recovery from 35.6 ± 8.4 to 10.4 ± 3.2 ms (*p* < 0.05, n = 4) (Figure 5B). Co-expression of p.L889V with WT channels produced very similar effects on the development (τ = 604 ± 89 ms and 0.45 ± 0.04) and recovery (τ = 13.2 ± 1.5 ms) from slow inactivation compared to the p.L889V variant alone.

**Figure 6:**
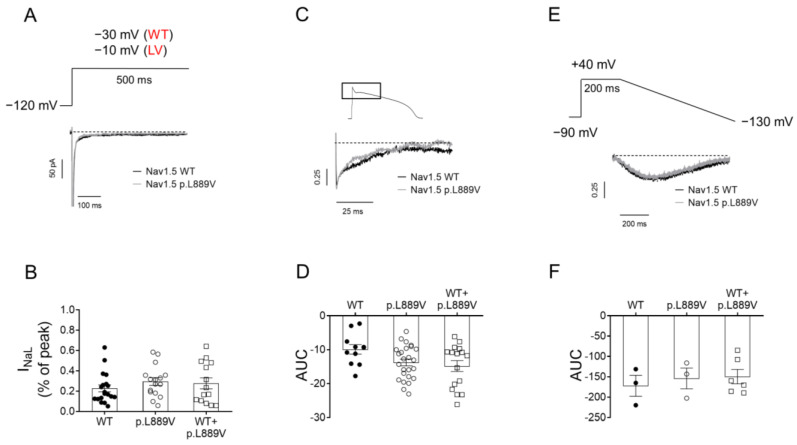
(**A**) I_Nav1.5_ traces recorded by applying 500 ms pulses from −120 mV to −30 or −10 mV in CHO cells transfected with the cDNA encoding WT or p.L889V Nav1.5, respectively. WT and LV: membrane potential when recording I_Na_ generated by WT and p.L889V channels, respectively. (**B**) Mean I_Na,L_ measured at the end of the 500 ms pulses and expressed as percentage of the peak current recorded in CHO cells transfected with the cDNA encoding WT (at −30 mV), p.L889V (at −10 mV), or WT and p.L889V (at −10 mV) Nav1.5. (**C**) Normalized current traces of the I_NaL_ recorded by applying a human ventricular AP command signal as voltage protocol in CHO transfected with the cDNA encoding WT or p.L889V Nav1.5. For clarity, an expanded view of traces (≈100 ms) shows the persistent inward Na current that deviates from the background current generated during the AP. (**D**) Mean AUC of the I_NaL_ traces recorded in the three experimental groups. (**E**) Normalized current traces of the I_NaL_ recorded by applying the ramp protocol shown at the top in CHO transfected with the cDNA encoding WT or p.L889V Nav1.5. (**F**) Mean AUC of the I_NaL_ traces recorded in CHO transfected with the cDNA encoding WT, p.L889V, or WT and p.L889V Nav1.5. In (**B**,**D**,**F**), bars represent the mean ± SEM of ‘n’ experiments, and each dot represents one single experiment. ANOVA followed by Tukey’s test and multilevel mixed-effects model.

To determine the possible effects on I_NaL_, we recorded it in CHO cells expressing the WT, p.L889V, or WT and p.L889V Nav1.5 using three different pulse protocols: 500 ms pulses from −120 to −30 (WT) or −10 mV (p.L889V and WT and p.L889V) (Figure 6A), pulses with the morphology of a human endocardial AP (Figure 6C), and 200 ms pulses from a holding potential of −90 to +40 mV followed by hyperpolarizing ramps to −130 mV (800 ms) (Figure 6E). The presence of the p.L889V variant alone or in combination with WT channels did not significantly modify the I_NaL_, regardless of the protocol used to measure it (Figure 6B,D,F).

### 2.3. Membrane Expression of p.L889V Channels

**Figure 7:**
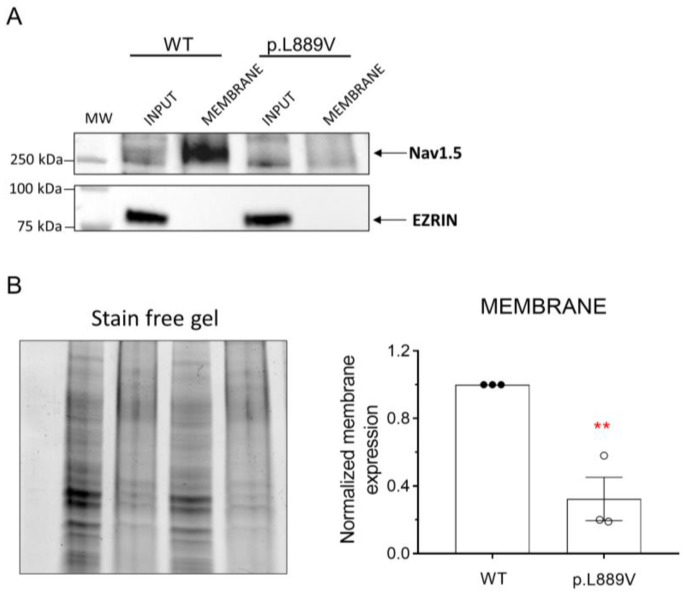
(**A**,**B**) Representative Western blot images (**A**) and densitometric analysis (**B**) of biotinylation assays showing the total (input) or surface (membrane) expression of WT and p.L889V Nav1.5 channels. The cytosolic protein ezrin was used as a negative control. In (**B**) (**left**), the corresponding stain-free gel is depicted to show the total protein. MW: molecular weight. In (**B**), bars show the mean ± SEM of 3 independent experiments. ** *p* < 0.01 vs. Nav1.5 WT. Unpaired student *t*-test.

The effects of the presence of the p.L889V variant on the membrane expression of Nav1.5 channels were tested using a cell surface biotinylation assay [11]. To this end, human embryonic kidney (HEK-293) cells were transfected with the cDNA encoding WT or p.L889V Nav1.5 channels, and the levels of total protein (inputs) and membrane expression were measured by Western blot (Figure 7A,B). The cytosolic protein ezrin was used as a negative control. Figure 7A,B shows a representative image depicting the total and membrane expression (biotinylated fractions) and densitometry of the relative Nav1.5 surface expression normalized to total protein. We did not detect significant differences in the expression of total Nav1.5 proteins between cells transfected with WT or p.L889V (Figure 7B). Conversely, the expression at the plasma membrane was significantly reduced in cells expressing the variant (Figure 7B). As expected, ezrin was not detected in the biotinylated extracts (Figure 7A).

### 2.4. Molecular Modeling

**Figure 8:**
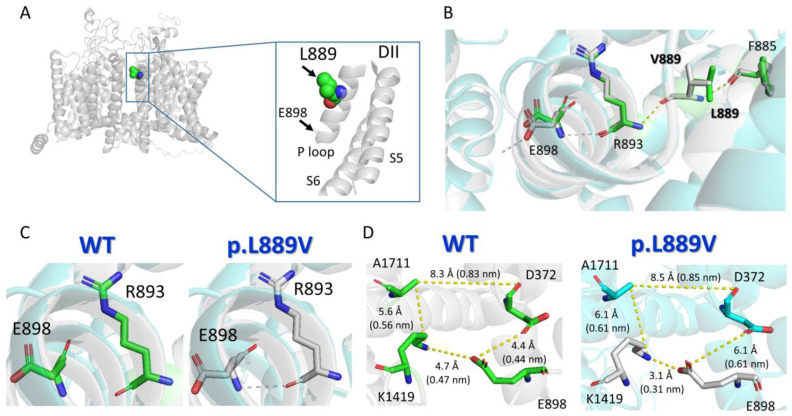
Molecular modeling of WT and p.L889V Nav1.5 channels. (**A**) *Top panel.* Ribbon representation of the cryo-EM structure of rat Nav1.5 (PDB: 6UZ3). The transmembrane segments (TM) are represented in gray, and the L889 residue has been highlighted. The inset shows a close view of the S5, P loop, and part of the S6 of DII. (**B**) Sticks representation of the amino acid side-chains from WT (green skeleton) and p.L889V (gray skeleton) showing the H-bonds established by the surrounding residues (F885, R863, and E898) in the presence of L889 (yellow) or V889 (gray). (**C**) Sticks representation of the R893 and E898 amino acids showing that in the presence of the valine ((**right**) panel), but not leucine ((**left**) panel), at position 889, a H-bond can be established between both (dashed line). (**D**) Sticks representation of the amino acid side-chains showing the distances (in Å and nm) among the residues of the DEKA motif (D372-E898-K1419-A1711) in WT Nav1.5 (L889, (**left**) panel) or in the presence of the mutation (V889, (**right**) panel). Throughout the figure, the numbering of the human Nav1.5 clone (NM_198056.3, Uniprot ID: Q14524) has been used.

Protein structure homology modeling was performed using the cryo-EM structure of rat Nav1.5 (PDB: 6UZ3) [12] and the SWISS-MODEL server (http://swissmodel.expasy.org accessed on 19 December 2023) [13] (Figure 8A). The molecular modeling suggested that the presence of the leucine-to-valine substitution at position 889 altered the conformation of various of the surrounding amino acids (Figure 8B), most notably the appearance of a slight torsion of the R893 that favors the establishment of an H-bond between R893 and E898 (Figure 8B,C). This H-bond cannot be established when the native amino acid (leucine) is the residue located at 889 (Figure 8C). As a consequence of these structural changes induced by the presence of the mutation, E898 is perturbed (Figure 8B,C), leading to a change in the distances to the residues (D372 and K1419) located in the equivalent positions of the P loop helix of DI and DIII domains (Figure 8D), which together with E898 and A1711 form the so-called DEKA motif of the selectivity filter (Supplementary Material).

## 3. Discussion

Here, we have clinically assessed the subjects belonging to three apparently unrelated Spanish families that share a rare *SCN5A* variant. Furthermore, we functionally analyzed p.L889V Nav1.5 channels and conducted in silico modeling to identify the possible determinants responsible for the variable penetrance and expressivity of the variant among carriers.

p.L889V channels alone or in combination with WT channels generate a reduced I_Na_ as a consequence of a marked decrease in expression at the cell membrane and a positive shift in the voltage dependence of activation. These effects would reduce the AP upstroke and intraventricular conduction velocity. The conduction disease that affects all carriers of this mutation belonging to family #3 (responsible for at least two SCDs) is thus attributable to the I_Na_ decrease. Along the same line, hampered conduction between the sinoatrial node and the atria could lead to SND. Thus, the reduction in I_Na_ can also underlie the SND observed in a carrier of the mutation belonging to family #2. Previous reports demonstrated that the greater the I_Na_ density reduction, the greater the penetrance of the BrS [14]. Therefore, the pronounced LOF produced by the mutation may explain the BrS exhibited by the proband of family #1 and a cousin of the proband of family #3.

Inactivation of Nav1.5 channels comprises fast and slow inactivation [10]. Our results demonstrated that p.L889V Nav1.5 channels recovered faster than WT from both types of inactivation. Furthermore, under physiological conditions, a small fraction of Nav1.5 channels remains open or reactivates prematurely during phase 3 of the AP, mediating the I_NaL_ and window current, respectively [10]. I_NaL_ generated by p.L889V channels was indistinguishable from that generated by WT channels. Moreover, p.L889V Nav1.5 channels activated and inactivated at more depolarized potentials than WT. These effects on the voltage dependence of channel activation and inactivation produced by the variant led to a slight decrease in the probability of the channels being partially but not fully inactivated, i.e., a slight decrease in the window current whose peak shifted to more depolarized potentials. Overall, our results demonstrate that the p.L889V variant disturbs the activation, inactivation, and recovery from inactivation of Nav1.5 channels, thus affecting all their distinctive gating properties. Interestingly, none of the changes produced by the variant would have led to an increased Na^+^ influx during the plateau phase of AP.

Our molecular modeling analysis suggests that the presence of the p.L889V Nav1.5 mutation alters the structural conformation of the nearest residues (e.g., R893) and that this change may be transmitted remotely to amino acids (E898) with a critical role in channel function located farther away by an allosteric mechanism. Thus, we propose that the reduction in I_Na_ produced by the p.L889V Nav1.5 mutation could be due, at least in part, to an alteration of the structural conformation of the P loop region that would eventually affect the channel pore.

In addition to the gating alterations, the expression levels of p.L889V Nav1.5 channels at the membrane are very low, which means that they hardly traffic being trapped into the endoplasmic reticulum, the Golgi apparatus, or both. Importantly, I_Na_ density and properties generated by p.L889V and WT channels are quite similar to that generated by p.L889V channels alone. These results suggest that the mutated channels act as “poison proteins” that concurrently trap native channels in intracellular compartments. As a consequence, mutated channels limit the traffic of WT channels toward the membrane, producing a marked DNE. Additionally, some Nav1.5 gating-deficient channels are able to transcomplementate, i.e., partially rescue I_Na_ by restoring the membrane expression of trafficking-deficient mutants when they are co-expressed together [15,16]. All these data led to the proposal that Nav1.5 channels not only traffic together but also form dimers at the membrane and that this physical interaction coupled their gating properties [17]. Following this hypothesis, DNE mechanisms not only involve trafficking deficiencies of mutant Nav1.5 channels but can also arise from the coupling of their altered gating properties [18]. However, other authors did not find any evidence of functional interactions or coupled gating, at least between Nav1.5 channels [19].

### Clinical Implications

The p.L889V Nav1.5 variant presents with an extremely low frequency in the general population (has not been annotated in ClinVar or gnomAD) and segregates within the affected families (particularly in family #3). Furthermore, our functional analysis confirmed that the mutation severely affects the biology and gating of Nav1.5 channels, leading to a significant decrease in I_Na_ (PS3: strong evidence of pathogenicity). Thus, the p.L889V variant can be classified as pathogenic, according to the ACMG guidelines [7]. The question is how the LOF modifications produced by the variant are reflected in the phenotype of the carriers.

As described previously with other LOF *SCN5A* variants, the mutation produces quite diverse phenotypic manifestations (BrS, conduction disease, DCM, and SND), which differ among different carriers and families (overlap syndrome) [4]. Moreover, these different phenotypic expressions are frequently found in combinations among the carriers of the same *SCN5A* variant within a family, as seen in affected subjects belonging to family #3. Similar results were previously described in two families carrying the p.E161K mutation. In this case, the clinical phenotype was highly variable in the affected subjects and manifested by different combinations of SND, conduction disease, and BrS [20].

BrS is probably linked to progressive histological or structural ventricular abnormalities. Although around 20 genes have been related to BrS, the association between them and the syndrome is very weak except for *SCN5A* [8,21]. Previous data confirmed that LOF mutations, particularly those producing DNE, confer an especially high burden of BrS [22]. From this point of view, results from functional studies of Nav1.5-mutated channels are of great value since they have therapeutic and prognostic implications [23]. Therefore, considering the marked LOF and DNE produced by the p.L889V variant, we would have expected that most carriers express an overt BrS phenotype. Conversely, among the 17 alive subjects harboring the mutation, only 2 have BrS (11%). Our results add further support to the contention that the relationship between I_Na_ reduction and clinical phenotype in *SCN5A* variants is inconsistent or weaker than proposed [24]. Thus, the incomplete penetrance of BrS suggests that additional factors are needed to elicit the BrS phenotype [4]. Growing evidence suggests that the concurrent presence of common and rare variants in other genes (genetic modifiers) could be responsible for the expressivity of BrS among carriers of a causative *SCN5A* variant [25]. Interestingly, the proband of family #2 presents two *SCN5A* LOF variants in compound heterozygosity; however, he does not present with a BrS. In addition to the genetic modifiers, sex and age are the most important factors in determining phenotypic expressivity. So, male sex predisposes to BrS, while female sex predisposes to conduction disease [2]. Conversely, among our probands, men actually present conduction defects, while the female proband was diagnosed with BrS.

It has been proposed that the differentiation between pathogenic or benign variants is insufficient, and complementary models predicting penetrance are necessary. Recently, a Bayesian approach was designed to predict the penetrance of *SCN5A* variants responsible for BrS, which is mainly based on variant-specific attributes such as the position of the substituted residue, sequence conservation, and the intensity of the functional alteration, together with observations of affected and unaffected heterozygotes from data available in public repositories and cohorts [26]. The p.L889V variant lays in the pore helixes of the second domain of the channel and, as mentioned, is extremely conserved among different species and within the four pore helix of the four Nav1.5 domains. It would have been very interesting to compare the BrS penetrance predicted by the Bayesian model for the p.L889V mutation with the actual penetrance in our families.

More than 60 genes, including *SCN5A*, have been related to DCM. *SCN5A*-related DCM exhibits an age-dependent penetrance with phenotypes at an increasing age and is commonly associated with severe conduction defects (that frequently precede DCM onset by 15 to 20 years) and arrhythmias, including AF and SND. In family #2, only the proband is positively diagnosed with DCM, and he is the single individual that carries both the p.L889V and the p.V1353M variants in compound heterozygosity. However, it is possible that as they grow older, other family members carrying the p.L889V variant may also manifest DCM, increasing the penetrance of the disease in this family. Similarly, other cases of patients with DCM and severe conduction disturbances have been published in subjects with *SCN5A* variants in compound heterozygosity (p.W156X and p.R225W and p.L256del and L1621F) [27,28].

Overall, these results reinforce the contention that incomplete penetrance of *SCN5A* mutations may result from a combination of multiple factors (I_Na_ reduction, localization of the mutation within the channel, concomitant presence of other common or uncommon genetic factors, and age and sex) and that, at present, it seems difficult to establish which of these factors determines penetrance the most. Thus, unfortunately, despite all efforts to identify more reliable predictive methods, it is still very difficult today to establish a genotype–phenotype relationship for new rare *SCN5A* variants.

## 4. Methods

### 4.1. Study Approval

The clinical evaluation of the probands and their relatives was approved by the Investigation Committee (PI 2023 04 1261) of the Hospital Universitario de Salamanca (Spain) and conforms to the principles outlined in the Declaration of Helsinki. Each participant gave written informed consent for clinical and genetic evaluation.

### 4.2. Clinical Study and Genetic Analysis

The probands and their relatives were clinically evaluated at the “Inherited Cardiovascular Disease Unit” in the Cardiology Department of the mentioned hospital.

The DNA was extracted from peripheral blood, and genetic testing was done using next-generation sequencing panels following the described procedures [29,30,31]. Variants identified (Table S1) were confirmed using the Sanger method and classified according to ACMG [7]. Other exonic non-synonymous variants found in probands of families #2 and #3 are indicated in Table S3. A phenotype–genotype segregation study was performed through cascade screening among available relatives of the three families with genetic study using Sanger sequencing [29,30,31].

### 4.3. cDNA Constructs, Mutagenesis, Cell Culture, and Transfection

CHO (for I_Nav1.5_ recordings) and HEK-293 (for biotinylation assays) cells were grown as previously described [11,32,33] and transiently transfected with the cDNA encoding human WT or p.L889V mutant Nav1.5 (NM_198056.2) (1.6 µg) and Navβ1 (NM_001037.4) (1.6 µg) subcloned in the pCGI vector kindly provided by Dr. Connie R. Bezzina (University of Amsterdam, Amsterdam, The Netherlands) [30,32,34]. The p.L889V Nav1.5 mutant was introduced using the QuikChange Site-Directed Mutagenesis kit (Agilent, Santa Clara CA, USA), and the variant was confirmed by direct DNA sequencing [30,31,32]. In some electrophysiological experiments, WT and p.L889V Nav1.5 were cotransfected (WT and p.L889V) in CHO cells at a 0.5:0.5 ratio (0.8 µg each).

### 4.4. Recording Techniques

I_Nav1.5_ was recorded at room temperature (21–23 °C) by means of the whole-cell patch-clamp technique using Axopatch-200B patch clamp amplifiers and pCLAMP 10.8 software (Molecular Devices, Palo Alto, CA, USA) [30,32,33]. Micropipette resistance was kept below 1.5 MΩ when filled with the internal solution and immersed in the external solution (see composition in Supplementary Materials). Under our experimental conditions, no significant voltage errors (<5 mV) due to series resistance were expected with the micropipettes used. The current amplitude measured in each experiment was normalized to membrane capacitance to obtain current density.

### 4.5. Biotinylation Assay

A biotinylation assay was conducted in HEK-293 cells using procedures previously described [11,31]. At the point of 48 h after transfection of WT or p.L889V, HEK-293 cells were biotinylated using EZ Link Sulfo-NHS-SS-Biotin (ThermoFisher Scientific, Waltham, MA, USA). After sample processing, Nav1.5 and ezrin proteins were detected by Western blot [11,31] using rabbit monoclonal anti-Nav1.5 (1:1000 Sigma S0819, St Louis, MI, USA) and mouse monoclonal anti-ezrin (1:400; ab4069 Abcam, Cambridge, UK) primary antibodies. The expression of the proteins in the biotinylated (membrane) fraction was normalized to the total protein expression.

### 4.6. Molecular Modeling

To determine the putative structural consequences of the p.L889V mutation, molecular modeling was performed using the cryo-EM structure of rat Nav1.5 (PDB: 6UZ3) and the protein structure homology modeling server, SWISS-MODEL (http://swissmodel.expasy.org, accessed on 19 December 2023) [13].

### 4.7. Statistics

The results are expressed as mean ± SEM. Statistical analysis was performed using GraphPad Prism 8 or Excel. To compare data from 3 experimental groups, a one-way ANOVA followed by Tukey’s test was used. An unpaired, two-sided *t*-test was chosen when comparing data from 2 experimental groups. In small-sized samples (n < 5), statistical significance was confirmed by using nonparametric tests (two-sided Wilcoxon’s test). To take into account repeated sample assessments, the data were analyzed with multilevel mixed-effects models. The normality assumption was verified using the Shapiro–Wilk test. Variance was comparable between groups throughout the manuscript. We chose the appropriate tests according to the data distributions. A value of *p* < 0.05 was considered significant. For the different groups of experiments, the sample size was chosen empirically according to previous experience in the calculation of experimental variability. No statistical method was used to predetermine the sample size. No particular procedure was followed for randomization/allocation of the respective experimental groups. Due to the nature of the experiments, the investigators were not blinded.

Additional methodological details are included in Supplementary Materials.

## 5. Conclusions

The p.L889V variant is pathogenic, and all the traffic and gating alterations that it produced justified the minimal I_Na_ generated by the combination of native and p.L889V Nav1.5 channels. The findings in these three families further illustrate the difficulty encountered in screening at-risk individuals, correlating genotype with phenotype, and predicting the course of disease in patients with novel, very rare *SCN5A* variants.

## Data Availability

The raw data supporting the conclusions of this article will be made available by the authors upon reasonable request.

## Supplementary Materials

The following supporting information can be downloaded at https://www.mdpi.com/article/10.3390/ijms25094686/s1. References [7,11,12,13,29,30,31,32,33,35,36,37] are cited in the Supplementary Materials.
