# Supplementary material for: Variable Penetrance and Expressivity of a Rare Pore Loss-of-Function Mutation (p.L889V) of Nav1.5 Channels in Three Spanish Families
Source: Int J Mol Sci. 2024 Apr 25;25(9):4686. doi: 10.3390/ijms25094686 (PMC11083067; doi:10.3390/ijms25094686)
Supplement: Supplementary file 1 [file ijms-25-04686-s001.zip › ijms-2913894-supplementary.pdf]

Variable penetrance and expressivity of a rare pore loss-of-function mutation (p.L889V) of Nav1.5 channels in three Spanish families

María Gallego-Delgado,[*1,2] Anabel Cámara-Checa,[*2,3] Marcos Rubio-Alarcón,[2,3] David Heredero-Jung,[4] Laura de la Fuente-Blanco,[1,2] Josu Rapún,[2,3] Beatriz Plata-Izquierdo,[5] Sara Pérez-Martín,[2,3] Jorge Cebrián,[2,3] Lucía Moreno de Redrojo,[1,2] Belén García-Berrocal,[4] Eva Delpón,[2,3] Pedro L Sánchez,[1,2] Eduardo Villacorta,[†1,2] Ricardo Caballero,[†2,3]

SUPPLEMENTARY MATERIAL

Supplementary Figures

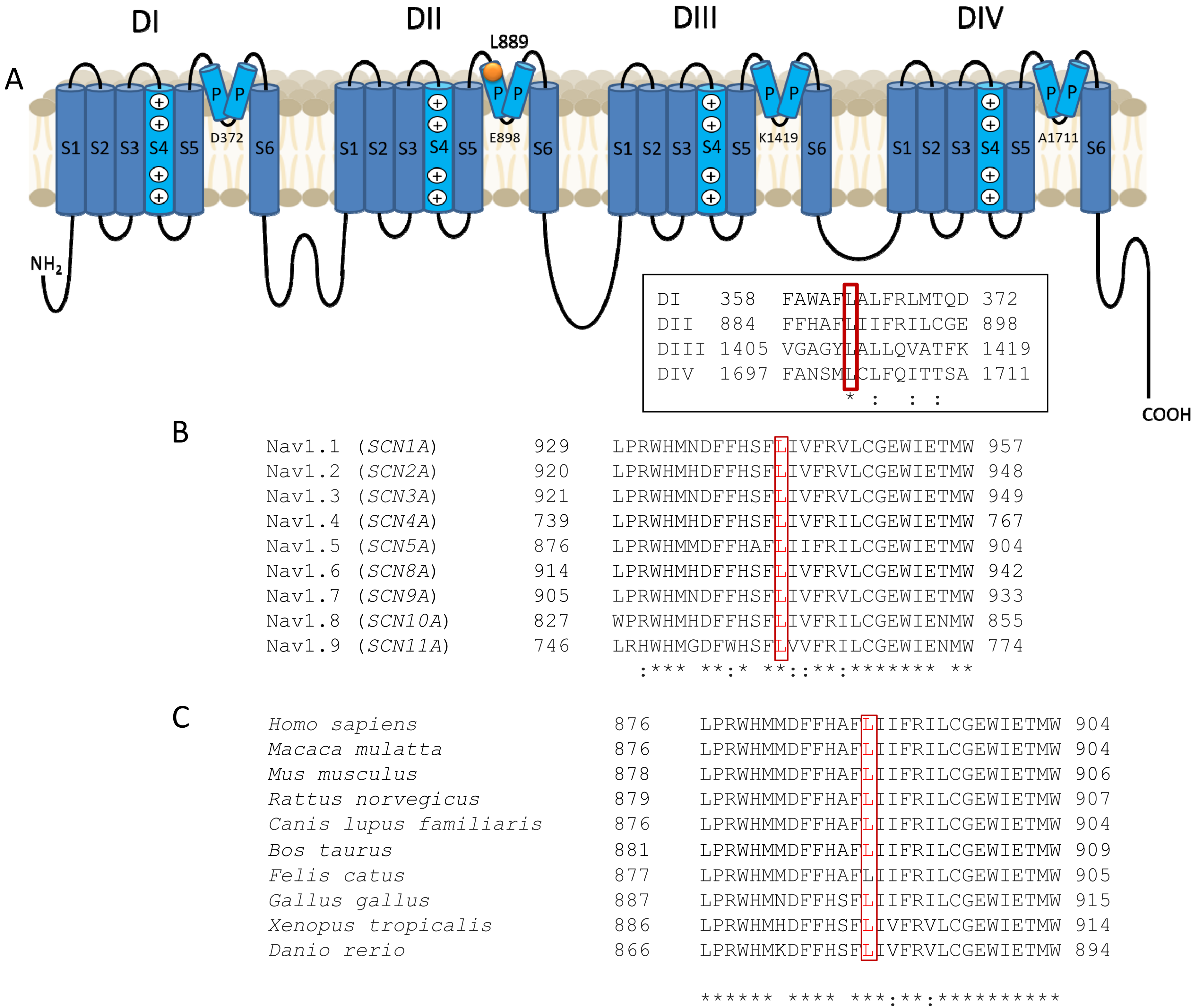
DI
DII
DIII
DIV
A
L889
P
S1
S2
S3
S4
S5
S6
D372
E898
K1419
A1711
NH2
COOH
DI 358 FAWAFLALFRLMTQD 372
DII 884 FFHAFLIIFRILCGE 898
DIII 1405 VGAGYLALLQVATFK 1419
DIV 1697 FANSMLCLFQITTSA 1711
* : : :
B
Nav1.1 (SCN1A) 929 LPRWHMNDFFHSFLIVFRVLCGEWIETMW 957
Nav1.2 (SCN2A) 920 LPRWHMHDFFHSFLIVFRVLCGEWIETMW 948
Nav1.3 (SCN3A) 921 LPRWHMNDFFHSFLIVFRVLCGEWIETMW 949
Nav1.4 (SCN4A) 739 LPRWHMHDFFHSFLIVFRILCGEWIETMW 767
Nav1.5 (SCN5A) 876 LPRWHMMDFFHAFLIIFRILCGEWIETMW 904
Nav1.6 (SCN8A) 914 LPRWHMHDFFHSFLIVFRVLCGEWIETMW 942
Nav1.7 (SCN9A) 905 LPRWHMNDFFHSFLIVFRVLCGEWIETMW 933
Nav1.8 (SCN10A) 827 WPRWHMHDFFHSFLIVFRILCGEWIENMW 855
Nav1.9 (SCN11A) 746 LRHWHMGDFWHSFLVVFRILCGEWIENMW 774
:*** **:* **::**:******* **
C
Homo sapiens 876 LPRWHMMDFFHAFLIIFRILCGEWIETMW 904
Macaca mulatta 876 LPRWHMMDFFHAFLIIFRILCGEWIETMW 904
Mus musculus 878 LPRWHMMDFFHAFLIIFRILCGEWIETMW 906
Rattus norvegicus 879 LPRWHMMDFFHAFLIIFRILCGEWIETMW 907
Canis lupus familiaris 876 LPRWHMMDFFHAFLIIFRILCGEWIETMW 904
Bos taurus 881 LPRWHMMDFFHAFLIIFRILCGEWIETMW 909
Felis catus 877 LPRWHMMDFFHAFLIIFRILCGEWIETMW 905
Gallus gallus 887 LPRWHMNDFFHSFLIIFRILCGEWIETMW 915
Xenopus tropicalis 886 LPRWHMHDFFHSFLIVFRVLCGEWIETMW 914
Danio rerio 866 LPRWHMKDFFHSFLIVFRVLCGEWIETMW 894
****** **** ***:**:**********

Figure S1. (A) Schematic diagram of human Nav1.5 channel (NM_198056.3, Uniprot ID: Q14524) indicating the approximate location of the L889 residue (DII) and of the aspartate (D372-DI), glutamate (E898-DII), lysine (K1419-DIII), and alanine (A1711-DIV) residues of the DEKA locus. Below panel A it is shown the alignment of the amino acids of the first helix of the P loop regions within the four domains of Nav1.5. **(B and C)** Sequence alignment of the region surrounding the L889 residue in nine channels of the Nav1.x family (**B**) and in Nav1.5 of several species (**C**). The red boxes highlight the conservation of the L889 amino acid, "*" means that the residues in the column are identical in all sequences in the alignment, and ":" indicates that the residues share strongly similar properties.

FAMILY #1

Proband 1III.1

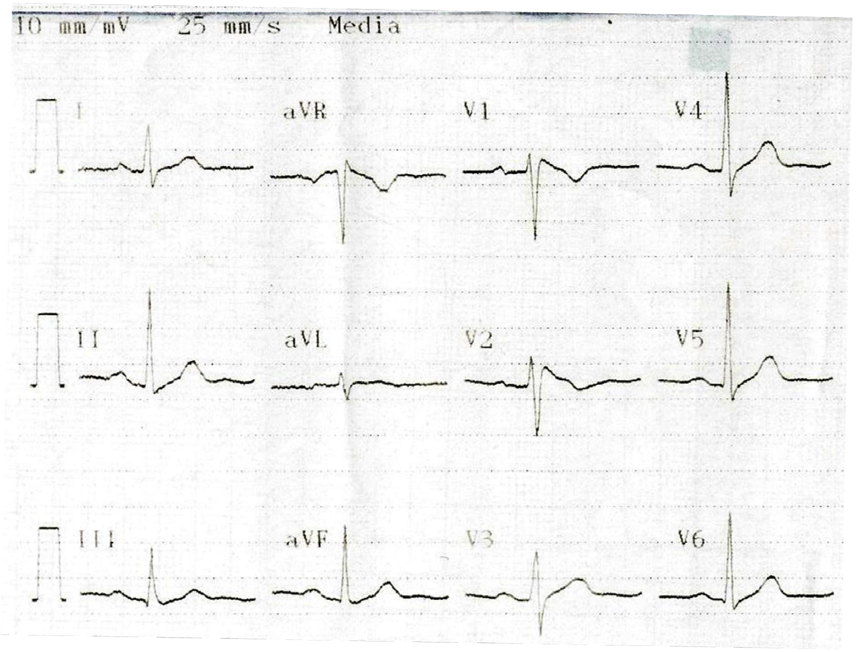

FAMILY #2

Proband 2III.2

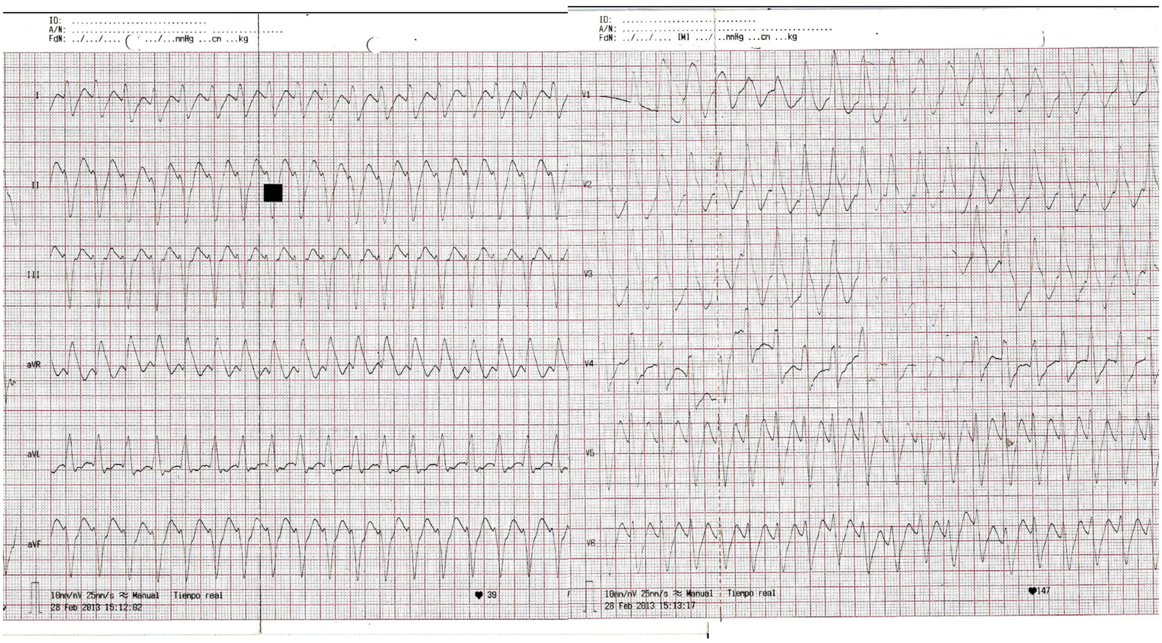

FAMILY #3

Proband 3IV.5

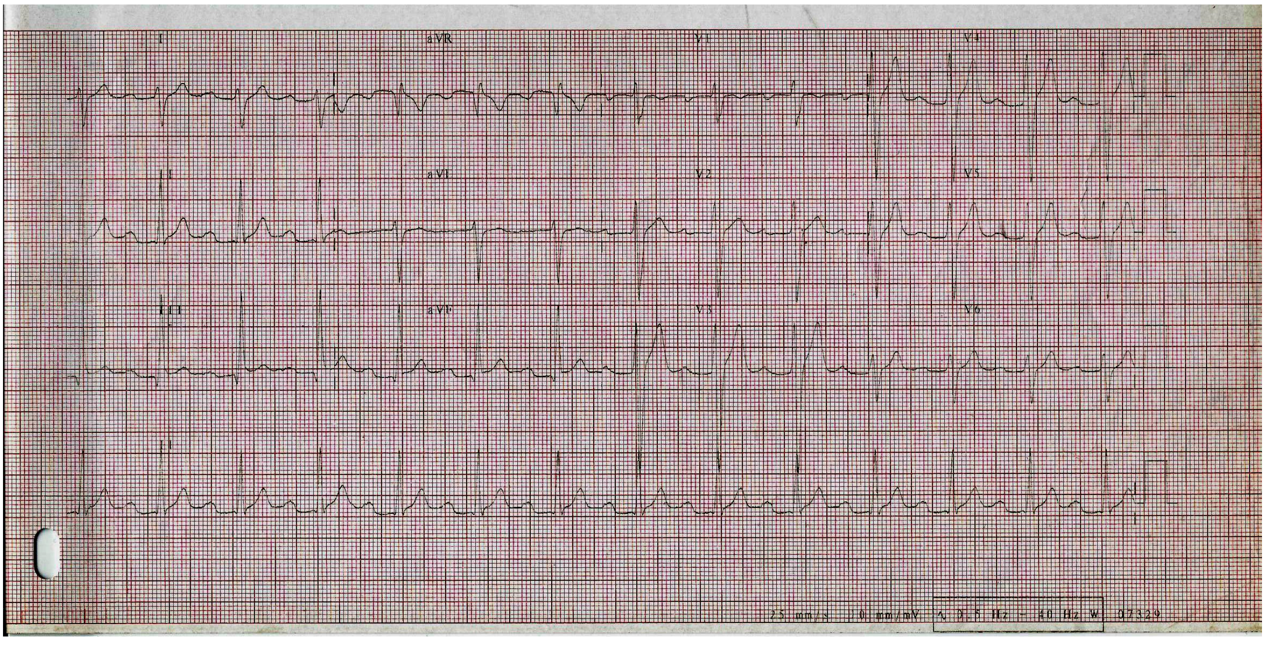

Figure S2. Twelve lead electrocardiogram (ECG) of the probands of family #1 (1III.1), #2 (2III.2), and #3 (3IV.5).

SUPPLEMENTARY MATERIAL

Supplementary Tables

Table S1. Summary of the prediction of pathogenicity of the Nav1.5 p.L889V (Families #1, #2, and #3) and p.V1353M (Family #2) variants.

Genotype	Genomic Location-GRCh37 (SNP ID)	Amino acid substitution	Pathogenicity predictors						
			Alphamissense	CADD Score	FATHMM	Mutation Taster	MutPred2	Polyphen-2 Prediction	SIFT
Heterozygous	3.38627304G>C (-)	L889V	Likely pathogenic (0.945)	24.7	Damaging	Disease causing	Pathogenic (0.810)	Probably damaging (0.965)	Deleterious
Heterozygous	3.38601826C>T (rs199473233)	V1353M	Likely pathogenic (0.828)	32	Damaging	Disease causing	Pathogenic (0.918)	Probably damaging (1.000)	Deleterious

Alphamissense= score ranges between 0 and 1, with a higher score indicating a greater propensity to be clinically pathogenic; CADD= Combined Annotation Dependent Depletion (A score greater of equal 20 indicates that it is predicted to be the 1% most deleterious substitutions that you can do to the human genome); FATHMM= Functional Analysis through Hidden Markov Models; MutPred2= score ranges between 0 and 1, with a higher score indicating a greater propensity to be pathogenic; Polyphen-2= score ranges from 0.0 (tolerated) to 1.0 (deleterious); SIFT= Sorting Intolerant From Tolerant. The variants shown are numbered according to the NM_198056.3 transcript reference sequence.

Table S2. Differential characteristics among carriers and non-carriers of the p.L889V variant. Subjects under 16 years (pediatric age) were excluded.

	Carriers	Non-Carriers	p value
**N**	13	8	NS
**Males**	5 (38%)	2 (25%)	NS
**Abnormal ECG**	8 (89%)	1 (12.5%)	0.067
**Age first abnormal ECG, years**	45±14	59±14	0.040
**PR, ms**	215±54	155±19	0.003
**QRS, ms**	117±42	90±20	NS
**First grade AVB**	7 (54%)	0 (0%)	0.018
**BFB (%)**	5 (38%)	0 (0%)	NS
**Age at first arrhythmia, years**	47±13	61±14	0.048
**AF/Aft (%)**	3(23%)	0 (0%)	NS
**Pacemaker (%)**	4 (19%)	0 (0%)	NS
**Brugada pattern (%)**	2 (15%)	0 (0%)	NS
**Ventricular arrhythmia (%)**	2 (15%)	0 (0%)	NS
**LVEF≤50%**	1 (8%)	0 (0%)	NS
**MACE**	2 (15%)	0 (0%)	NS

AF: atrial fibrillation; Aft: atrial flutter; AVB: atrioventricular block; BFB: bifascicular block; ECG: electrocardiogram; LVEF: left ventricular ejection fraction; MACE: major adverse cardiovascular events including sudden cardiac death, sustained ventricular arrhythmia, heart failure or death; NS: not significant.

Table S3. Summary of other nonsynonymous variants identified in the probands of families #2 and #3.

Gene	Variant	dbSNP_ID	MAF	Aminoacid substitution	Transcript
		FAMILY #2			
ALMS1	NC_000002.11:g.73679866T>C	rs10496192	0.1585	I2070T	NM_015120.4
ALMS1	NC_000002.11:g.73679956C>T	rs28730854	0.03052	S2100L	NM_015120.4
BAG3	NC_000010.10:g.121436286C>T	rs3858340	0.1008	P407L	NM_004281.3
FLNC	NC_000007.13:g.128488734G>A	rs2291569	0.08189	R1567Q	NM_001458.4
HFE	NC_000006.11:g.26091179C>G	rs1799945	0.1320	H63D	NM_000410.3
OBSCN	NC_000001.10:g.228432264A>T	rs41305731	0.03664	H1158L	NM_001098623.2
OBSCN	NC_000001.10:g.228486404C>T	rs56021350	0.1749	T4399M	NM_001098623.2
OBSCN	NC_000001.10:g.228496066G>T	rs55704206	0.03331	A4241S	NM_001098623.2
OBSCN	NC_000001.10:g.228505739G>A	rs3795801	0.1770	G4666S	NM_001098623.2
OBSCN	NC_000001.10:g.228525008G>A	rs3795809	0.1286	*R5574H*	NM_001098623.2
PKP2	NC_000012.11:g.33021934A>G	rs1046116	0.2146	*L366P*	NM_004572.3
SPEG	NC_000002.11:g.220333321G>A	rs35181232	0.01095	*R1055Q*	NM_005876.4
RYR2	NC_000001.10:g.237586384T>C	rs16835237	0.1415	*Intron (splicing region)*	NM_001035.2
SYNE1	NC_000006.11:g.152757224G>A	rs34028822	0.003887	*R1388W*	NM_182961.3
TMPO	NC_000012.11:g.98927830C>G	rs17459334	0.09178	*Q599E*	NM_003276.2
TTN	NC_000002.11:g.179650408G>A	rs35813871	0.1687	*T765I*	NM_003319.4
TTN	NC_000002.11:g.179498042T>C	rs6723526	0.09758	*K11752E*	NM_003319.4
TTN	NC_000002.11:g.179393859A>G	rs55880440	0.01573	*I26475T*	NM_003319.4

Gene	Variant	dbSNP_ID	MAF	Aminoacid substitution	Transcript
		FAMILY #3			
AKAP9	NC_000007.13:g.91708898A>G	rs35759833	0.1264	*K2484R*	NM_005751.4:
DSG2	NC_000018.9:g.29122618G>A	rs79241126	0.06741	*E773K*	NM_001943.3
DSG2	NC_000018.9:g.29100761G>T	rs80073511	0.002077	*Intron (splicing región)*	NM_001943.3
GAA	NC_000017.10:g.78087041G>A	rs1800309	0.04552	*E689K*	NM_000152.3
KCNA5	NC_000012.11:g.5154242C>T	rs17215402	0.006717	*P310L*	NM_002234.3
SCN5A	NC_000003.11:g.38647642G>T	rs41312433	0.1798	*Intron (splicing región)*	NM_198056.2
SNTA1	NC_000020.10:g.32026826C>T	rs75025585	0.001969	*R106Q*	NM_003098.2
SYNE1	NC_000006.11:g.152720912G>T	rs142747430	0.0001351	*T2366N*	NM_033071.3
TRDN	NC_000006.11:g.123696766G>T	rs17737379	0.1237	*D419E*	NM_006073.3
TTR	NC_000018.9:g.29172865G>A	rs1800458	0.06688	*G26S*	NM_000371.3

dbSNP: Single Nucleotide Polymorphism Database; MAF= mean minor allele frequency from all ethnic groups where the variant has been identified as provided in gnomad (https://gnomad.broadinstitute.org/). Only SNPs with a MAF less than 0.20 are shown. All these variants were predicted as benign or likely tolerated. The variants shown are numbered according to the transcript reference sequence indicated in the rightmost column.

SUPPLEMENTARY MATERIAL

Supplementary Methods

1. *Next-generation sequencing.*

The genetic analysis of the probands was conducted by using a next-generation sequencing panel including 96 genes (Health in Code, SL, Spain) following procedures previously described [1–3]. Genomic DNA was extracted using an automated extraction and purification process by using the QIAsymphony SP® kit (Qiagen, Hilde, Germany) according to the manufacturer's instructions. Library preparation was carried out using SureSelect Reagent kit (Agilent, Santa Clara, CA, USA) for Illumina's paired-end multiplexed sequencing method, following the manufacturer's instructions. The enrichment of the regions of interest was performed by means of a SureSelect (Agilent) probe kit that selectively captures the coding zones and the flanking intronic areas of the selected genes. After the generation of clusters, the DNA libraries were sequenced on the Illumina HiSeq 1500 platform. The analysis of the sequencing data was performed using a proprietary bioinformatics pipeline to obtain a report of variants noted along with their coverage and corresponding quality parameters. We excluded variants located in introns (except those in suspected splicing sites) or intergenic regions. We also removed synonymous variants and non-synonymous variants with occurrences >1 in our local database. Genetic variants considered clinically relevant and regions of low coverage in priority genes were re-sequenced in parallel using the Sanger method. The analytical sensitivity and specificity of this test are over 99% for single nucleotide substitutions (SNVs) and small insertions/deletions (INDELs). The list of variants identified was evaluated against database information on previously described variants [Human Gene Mutation Database (http://www.hgmd.cf.ac.uk/), Single Nucleotide Polymorphism (SNP) database (http://www.ncbi.nlm.nih.gov/SNP/), NHLBI GO Exome Sequencing Project (http://evs.gs.washington.edu/EVS), ClinVar (https://www.ncbi.nlm.nih.gov/clinvar/), or Genome Aggregation database (http://gnomad.broadinstitute.org). Pathogenicity of the identified variants was established according to the current recommendations of the American College of Medical Genetics and Genomics and the Association for Molecular Pathology [4]. Variant pathogenicity was graded according to its presence in a previously associated or candidate gene and the *in silico* predicted impact on the protein using widely used software (Alphamissense, Combined Annotation Dependent Depletion-CADD, Functional Analysis through Hidden Markov Models- FATHMM, MutPred2, Polyphen-2, and Sorting Intolerant From Tolerant-SIFT), the degree of conservation of the affected residue measured by multiple ortholog alignment using Alamut software (http://www.interactive-biosoftware.com).

2. *Sanger sequencing.*

A phenotype-genotype segregation study was conducted through cascade screening among available relatives with Sanger genetic study [1–3]. The study included the analysis of genomic positions where variants have previously been identified in a family member. PCR products were purified using illustra ExoProStar 1-Step (GE Healthcare Life Sciences, Chicago, IL, USA). PCR amplification was carried out using specific primers that were previously validated. After purification, the amplified fragments were sequenced from both ends. Genotype determination is performed using Variant Reporter software (Life Technologies). The results were compared with the reference sequence from GRCh37/hg19 by means of Chromas Lite Software (http://technelysium.com.au).

3. ***cDNA constructs and site-directed mutagenesis.***

Human cardiac Nav1.5 (hH1; NM_198056.2) and Navβ1 (NM_001037.4) cDNA subcloned in the pCGI vector were kindly gifted by Dr. Connie R. Bezzina (University of Amsterdam, The Netherlands). The p.L889V Nav1.5 mutant was made by using the QuikChange Site-Directed Mutagenesis kit (Stratagene, USA) and the variant was confirmed by direct DNA sequencing [1–3,5].

4. ***Cell lines used in the functional study and culture conditions.***

The study has been conducted in Chinese Hamster Ovary cells (CHO) and human embryonic kidney 293 cells (HEK293) purchased from American Type Culture Collection (ATCC, Manassas, VA, USA). They had been authenticated by the supplier as appropriate. Mycoplasma tests were conducted routinely and showed no mycoplasma contamination. CHO cells were grown in 60 mm dishes at 37ºC in an 5% CO_2 atmosphere and in Ham-F12 medium supplemented with 10% fetal bovine serum, 100 U/ml penicillin, and 100 μg/ml streptomycin [1,5,6,3]. HEK293 cells were cultured in 60 mm dishes at 37ºC in an 5% CO_2 atmosphere and in Dulbecco's modified Eagle's (DMEM) medium supplemented with 10% fetal bovine serum, 100 U/ml penicillin, and 100 μg/ml streptomycin as previously described [3,7]. The cultures were passaged every 4–5 d using a brief trypsin treatment.

5. Cell transfection.

Nav1.5 current ($I_{Nav1.5}$) was recorded in subconfluent cultures of CHO cells transiently transfected with the cDNA encoding WT or mutated Nav1.5 channels (1.6 μg) and hNavβ1 (1.6 μg) (Nav1.5-β1) plus the cDNA encoding the CD8 antigen (0.5 μg) by using X-tremeGENE™ HP (Roche Diagnostics, Rotkreuz, Switzerland), with manufacturer instructions followed [1–3,5–7]. In some experiments, WT and p.L889V Nav1.5 were cotransfected (WT+p.L889V) at a 0.5:0.5 ratio (0.8 μg each). In all cases, at the point of 48 h after transfection, cells were incubated with polystyrene microbeads precoated with anti-CD8 antibody (Dynabeads M450, Thermofisher Scientific). Most

of the cells that were beaded also exhibited channel expression. The day of recordings, CHO cells were removed from the dish by using a cell scraper and the cell suspension was stored at room temperature and used within 12 h for electrophysiological experiments. Biotinylation assays were conducted in HEK293 cells transfected with the same amount of cDNA encoding WT or mutated Nav1.5 channels (1.6 μg) and hNavβ1 (1.6 μg) than in CHO cells by using Lipofectamine 2000 (Thermofisher Scientific, Waltham, MA, USA) [3,7].

6. *Recording techniques.*

6.1. $I_{Nav1.5}$ recordings.

A small aliquot of the CHO cell suspension was placed in a 0.5 mL chamber mounted on the stage of an inverted microscope (Nikon TMS; Nikon Co., Tokio, Japan). After settling to the bottom of the chamber, cells were perfused at ≈1 mL/min with external solution (see the composition below). Macroscopic currents were recorded at room temperature (21-23ºC) by means of the whole-cell patch-clamp technique using Axopatch-200B patch clamp amplifiers and pCLAMP10 software (Molecular Devices, San José, CA, USA) [2,3,5–7]. Recording pipettes were pulled from 1.0 mm o.d. borosilicate capillary tubes (GD1, Narishige Co., Ltd, Tokio, Japan) using a programmable patch micropipette puller (Model P-2000 Brown-Flaming, Sutter Instruments Co., Novato, CA, USA) and were heat-polished with a microforge (Model MF-83, Narishige). Micropipette resistance was kept below 1.5 MΩ when filled with the internal solution and immersed in the external solution. In all the experiments, series resistance was compensated manually by using the series resistance compensation unit of the Axopatch amplifier, and ≥80% compensation was achieved. The remaining access resistance after compensation and cell capacitance were 1.4±0.3 MΩ and 13.3±1.1 pF (n=64), respectively. Therefore, under our experimental conditions no significant voltage errors (<5 mV) due to series resistance were expected with the micropipettes used. To minimize the contribution of time-dependent shifts of channel availability during $I_{Nav1.5}$ recordings, all data were collected 5-10 min after establishing the whole-cell configuration. Under these conditions current amplitudes and voltage dependence of activation and inactivation were stable during the time of recordings [2,5,6]. $I_{Nav1.5}$ were sampled at 50 kHz, filtered at half the sampling frequency and stored on the hard disk of a computer for subsequent analysis. To minimize the influence of the expression variability, each construct was tested in a large number of cells obtained from at least 3 different transfection batches. To record $I_{Nav1.5}$, CHO cells were superfused with an external solution containing (mM): NaCl 136, KCl 4, $CaCl_2$ 1.8, $MgCl_2$ 1, HEPES 10, and glucose 10 (pH 7.4 with NaOH), while recording pipettes were filled with an internal solution containing (mM): NaF 10, CsF 110, CsCl 20, HEPES 10, and EGTA 10 (pH 7.35 with CsOH).

6.2. Pulse protocols and analysis [1,2,5,6].

To construct the current-voltage relationships, 50-ms pulses in 5 mV increments from −120 mV to potentials between −80 and +50 mV were applied. On each experiment, the current amplitude was normalized to the membrane capacitance to obtain the current density. In order to describe the time course of $I_{Nav1.5}$ decay, a biexponential analysis was used as an operational approach, fitting peak current decay with an equation of the form:

$$y=C+A_f*\exp(-t/\tau_f)+A_s*\exp(-t/\tau_s)$$

where τ_f and τ_s are the fast and slow time constants, while A_f and A_s are the amplitudes of each component of the exponential, and C is the baseline value. To quantify the time course of current activation, a monoexponential function was fitted to the activation phase of currents generated by pulses to −10 mV yielding the time constant (τ_{act}) that defines the process. Conductance-voltage curves were constructed by plotting the normalized conductance as a function of the membrane potential. The conductance was estimated for each experiment by the equation:

$$G=I/(V_m-E_{rev})$$

where G is the conductance at the test potential V_m, I represents the peak maximum current at V_m, and E_{rev} is the reversal potential. To determine the E_{rev}, I_{Na} density-voltage relationships obtained in each experiment were fitted to a function of the form:

$$I=(V_m-E_{rev})*G_{max}*(1+\exp[V_m-V_h]/k)^{-1}$$

where I is the peak current elicited at the test potential V_m, G_{max} is the maximum conductance, and *k* is the slope factor.

To construct the inactivation-availability curves, the $I_{Nav1.5}$ was recorded by applying 500-ms pulses from −120 mV to potentials between −140 and −20 mV in 10 mV increments followed by a test pulse to −30 mV (WT) or −10 mV (p.L889V and WT+p.L889V). Inactivation curves were constructed by plotting the current amplitude recorded with the test pulse as a function of the membrane potential of the preceding pulse. A Boltzmann function was fitted to activation/conductance-voltage and inactivation curves to obtain the midpoint (V_h) and the slope (*k*) values of the curves. The window currents are represented as the enlarged portion of the

overlapping area between the activation and inactivation curves. We also calculated the probability of being within this window using the following equation:

$$(1/\{1+\exp[(V_{hact}-V)/k_{act}]\}X((1-C)/\{1+\exp[(V-V_{hinact})/k_{inact}]\}+C),$$

where V_{hact}, V_{hinact}, k_{act} and k_{inact} are the mean midpoint and slope values of the activation and inactivation curves and C is the bottom value of the fit of the inactivation curves with the Boltzmann equation [2].

To analyze the recovery from fast inactivation, two 50-ms pulses (P1 and P2) from –120 to −30 mV (WT) or -10 mV (p.L889V and WT+p.L889V) were applied at increasing coupling intervals (0.1-500 ms). A monoexponential function was fitted to the data to measure the reactivation kinetics.

The development of slow inactivation was evaluated by applying a prepulse (P1) from –120 to −30 mV (WT) or −10 mV (p.L889V and WT+p.L889V) of increasing duration (10-2000 ms) followed by a test pulse (P2) to −30 mV (WT) or −10 mV (p.L889V and WT+p.L889V). Between both pulses, a 20-ms pulse to –120 mV was applied to allow most channels to recover from fast inactivation. A monoexponential function was fitted to the data to obtain the time constant that defines the process. To analyze the recovery from slow inactivation, a 500-ms pulse (P1) from –120 to −30 mV (WT) or −10 mV (p.L889V and WT+p.L889V) was applied followed by a pulse to −120 mV of increasing duration (1.2-10000 ms) and a test pulse (P2) to −30 mV (WT) or −10 mV (p.L889V and WT+p.L889V). A biexponential function was fitted to the data to measure the kinetics of slow inactivation recovery.

I_{NaL} was recorded by applying 500-ms pulses from −120 to −30 mV (WT) or −10 mV (p.L889V and WT+p.L889V), measured as the leak-subtracted inward current remaining at the end of the pulse and represented as the ratio (in %) between late and peak I_{Na}. I_{NaL} was also recorded by applying a human endocardial action potential as the command signal. Using this protocol, an initial large inward current (peak I_{Na}) was recorded driven by the depolarizing phase of the action potential. During the repolarization phase, a background current that resembles the action potential morphology because it is almost linear to the voltage at this range of membrane potentials is superimposed on top of a small inward current (I_{NaL}) [2]. Therefore, the I_{NaL} was considered as the inward component that deviates from the background current and, in each experiment, it was measured as the area of the inward current that had been subtracted from the background current [2]. Finally, I_{NaL} was also recorded by applying 200-ms pulses from a holding

potential of −90 mV to +40 mV followed by hyperpolarizing ramps to −130 mV (800 ms). During the ramp development, a background current almost linear to the voltage at this range of membrane potentials is superimposed on top of a small inward current (I_{NaL}). Therefore, the I_{NaL} was considered as the inward component that deviates from the background current and, in each experiment, it was measured as the area of the inward current that had been substracted from the background current.

7. *Biotinylation assay.*

A biotinylation assay was conducted using previously described procedures [3,7] to determine putative changes in the membrane expression of Nav1.5 channels induced by the presence of the p.L889V mutation. At the point of 48 h after transfection of Nav1.5 WT or p.L889V channels, HEK293 cells were washed twice with ice cold phosphate-buffer saline (PBS) and biotinylated for 25 min at 4°C using PBS containing 0.5 mg/mL of EZ Link Sulfo-NHS-SS-Biotin (Thermo Scientific, USA). Plates were incubated for 10 min at 4°C with PBS-100 mM glycine (to quench unlinked biotin) and washed three times with PBS. Cells were collected in RIPA buffer containing 50 mM Tris·HCl (pH=7.5), 150 mM NaCl, 1% Nonidet P-40, 0.1% SDS, 0.5% sodium deoxycholate, and 1 mM phenylmethylsulfonyl fluoride (PMSF) and protease inhibitor cocktail (Sigma-Aldrich Inc.) for protein extraction. Subsequently, the extract (2 mg) was incubated with Streptavidin Sepharose (50 µl, Cytiva, USA) overnight at 4ºC. To separate biotinylated fraction, samples were centrifugated at 3000 rpm for 2 min at 4°C. The fraction of biotinylated proteins was washed by several centrifugations prior Western blot analysis. Detection of WT or p.L889V Nav1.5 channels and ezrin proteins was carried out by Western blot following previously described procedures [3,7]. Nuclei and cell debris were removed by centrifugation at 14.000 rpm for 20 min at 4ºC. The total protein amount of the extracts was calculated with the bicinchoninic acid method (Pierce). Samples were run on 4-15% Mini-PROTEAN TGX™ stain-free gels (Bio-Rad, USA) and, afterwards, protein was transferred to nitrocellulose membranes. Nonspecific binding sites were blocked with 5% non-fat dried milk in PBS with Tween-20 (0.05%) for 1 h at room temperature. Membranes were then incubated with rabbit monoclonal anti-Nav1.5 (1:1000 Sigma S0819) and mouse monoclonal anti-ezrin (1:400; ab4069 Abcam, UK) primary antibodies overnight at 4ºC. Afterwards, samples were incubated for 1 h with fluorescent-dye conjugated secondary antibodies (anti-rabbit HRP 1:10000, 111-035-144 or anti-mouse HRP 1:10000, 115-035-003. Jackson Immunoresearch, USA). Membranes were washed three times with PBS-Tween after adding primary and secondary antibodies. Protein expression was visualized by using the LED imaging modules of the Chemidoc MP System and Image Lab 5.2.1 software (Bio-Rad). Expression of the proteins in the biotinylated (membrane) fraction was normalized to the total protein expression.

8. Molecular modeling.

To determine putative structural consequences of the p.L889V mutation, a molecular modeling was performed using the cryo-EM structure of rat Nav1.5 (PDB: 6UZ3) fetched from Protein Data Bank (http://www.rcsb.org/pdb/). The p.L892V (rat clone numbering) was introduced by *in silico* mutagenesis and the mutated amino acid sequence was uploaded to the fully automated protein structure homology-modeling server, Swiss-model (http://swissmodel.expasy.org) [8], to build the p.L892V model using WT Nav1.5 PDB as a template. When the model running was completed, the software provided different conformations and the optimal conformation of the mutated form was chosen and refined using ModRefiner web server (https://seq2fun.dcmb.med.umich.edu//ModRefiner). Thereafter, WT and p.L892V Nav1.5 were compared on PyMOL (Molecular Graphics System, Version 2.0 Schrödinger, LLC) and putative changes in the amino acid positions, distances, and interactions (Hydrophobic, electrostatic and H-Bonds) with surrounding residues induced by the presence of the mutation were identified.

8.1. Extended results and discussion of the molecular model:

To preliminary predict putative structural consequences of the p.L889V mutation, we used Cupsat (Cologne University Protein Stability Analysis Tool; https://cupsat.brenda-enzymes.org/). Cupsat predicted that the mutation would reduce the overall stability of the protein and the energy in the most stable conformation (–1.71 kcal/mol) and change the torsion properties of the residue. Besides, the molecular modeling performed by using the Swiss-model server suggested that the presence of the leucine-to-valine substitution at position 889 altered the conformation of various of its surrounding amino acids, as can be deduced by the subtle changes in the distances between the residue at 889 and F885 or R893 (both located at less than 3 Å from 889). Moreover, the presence of the mutation produced a slight torsion of the R893 that favors the establishment of a H-bond between R893 and E898. Importantly, this H-bond cannot be established when the native amino acid (leucine) is the residue located at 889. As a result of all these structural changes, the model suggests that E898 disposition is markedly perturbed when the residue at position 889 changes from leucine to valine. E898 is located at the end of the P loop helix where, together with the residues (D372, K1419, A1711) at the equivalent positions of the other three P loop regions of domains I, III, and IV, respectively, forms the so-called DEKA motif of the selectivity filter [9]. Our modeling suggests that the p.L889V-induced effect over E898 leads to a remarkable alteration in the structural disposition of this region as a consequence of a change in the distances among the DEKA residues. In 2020, Jiang and coworkers described the high-resolution structure of the rat cardiac Nav1.5 channel at the atomic level [9] demonstrating that the four DEKA residues forms a nearly 4-fold symmetrical square whose disposition determines channel permeation and selectivity [9]. Therefore, it can be speculated that the presence of the p.L889V

mutation affects the symmetry of the selectivity filter contributing to reduce Na^+ flux through the ion channel. Strikingly, the functional analysis did not show any modification of the ion selectivity. Although the reasons for this result are unknown, it could be related to the different structural disposition and relative importance of the DEKA residues on ion selectivity. Indeed, D372 and A1711 line the walls of the ion-selectivity filter, whereas E898 and K1419 do not [9]. Furthermore, these authors proposed that K1419 is the main determinant of a high Na^+ selectivity [9]. Interestingly, results similar to ours were obtained with the p.S1710L mutation that affects a serine adjacent to the A1711 of the DEKA motif and whose functional analysis showed that it reduced I_{Na} density without modifying selectivity [10]. Thus, we propose that the introduction of valine at position 889 alters the structural conformation of the nearest residues and that this change may be transmitted remotely to amino acids located farther away through and allosteric mechanism. There are multiple examples in the literature of such changes in protein structure/function induced by point missense mutations mediated by allosteric mechanisms. Although the closer a residue is to the mutated amino acid, the greater the expected degree of perturbation, distant residues are frequently perturbed. Regarding Nav1.5 channels and SCN5A mutations, it is worth mentioning the p.E1784K Nav1.5 mutation identified in congenital long QT syndrome patients [11]. These authors demonstrated that substitution of a conserved glutamic acid located at the C-terminal domain by lysine severely affected channel function by an allosteric rather than a direct effect on channel gating [11]. Regarding other channels different from Nav1.5 and as a representative example, our group has recently characterized the gain-of-function p.V240M HCN4 mutation responsible for inappropriate sinus tachycardia in a Spanish family [3]. The variant, which lies in the N-terminal HCN domain (HCND), increased the density of the macroscopic current as well as single-channel conductance and opening frequency and probability of HCN4 channels. In that case the presence of the mutation produced an allosteric effect that eventually weakened the interaction between the HCND and the voltage sensor domain leading to strong effects on the biophysical properties of the channel despite the residue affected by the mutation was considerably separated from the channel pore [3]. In this context, it has been recently described a general rule for the extent of the perturbations caused by missense mutations together with a simple theoretical framework for modeling their allosteric effects [12]. Using molecular dynamics and ubiquitin as a model, these authors proposed that mutational effects consistently propagate beyond (even up to 15−20 Å) the immediate mutational neighborhood and dissipate exponentially with a decay distance constant of~4−5 Å [12]. Considering all of the above, it seems reasonable to propose that the reduction of the I_{Na} produced by the p.L889V Nav1.5 mutation could be due, at least in part, to an alteration of the structural conformation of the P loop region that would eventually affect channel pore.

Supplementary References

1. Caballero, R.; Utrilla, R.G.; Amorós, I.; Matamoros, M.; Pérez-Hernández, M.; Tinaquero, D.; Alfayate, S.; Nieto-Marín, P.; Guerrero-Serna, G.; Liu, Q.-H.; et al. Tbx20 Controls the Expression of the KCNH2 Gene and of hERG Channels. *Proc. Natl. Acad. Sci. U.S.A.* **2017**, *114*, E416–E425, doi:10.1073/pnas.1612383114.
2. Nieto-Marín, P.; Tinaquero, D.; Utrilla, R.G.; Cebrián, J.; González-Guerra, A.; Crespo-García, T.; Cámara-Checa, A.; Rubio-Alarcón, M.; Dago, M.; Alfayate, S.; et al. Tbx5 Variants Disrupt Nav1.5 Function Differently in Patients Diagnosed with Brugada or Long QT Syndrome. *Cardiovasc Res* **2022**, *118*, 1046–1060, doi:10.1093/cvr/cvab045.
3. Cámara-Checa, A.; Perin, F.; Rubio-Alarcón, M.; Dago, M.; Crespo-García, T.; Rapún, J.; Marín, M.; Cebrián, J.; Gómez, R.; Bermúdez-Jiménez, F.; et al. A Gain-of-Function HCN4 Mutant in the HCN Domain Is Responsible for Inappropriate Sinus Tachycardia in a Spanish Family. *Proc Natl Acad Sci U S A* **2023**, *120*, e2305135120, doi:10.1073/pnas.2305135120.
4. Richards, S.; Aziz, N.; Bale, S.; Bick, D.; Das, S.; Gastier-Foster, J.; Grody, W.W.; Hegde, M.; Lyon, E.; Spector, E.; et al. Standards and Guidelines for the Interpretation of Sequence Variants: A Joint Consensus Recommendation of the American College of Medical Genetics and Genomics and the Association for Molecular Pathology. *Genet Med* **2015**, *17*, 405–424, doi:10.1038/gim.2015.30.
5. Pérez-Hernández, M.; Matamoros, M.; Alfayate, S.; Nieto-Marín, P.; Utrilla, R.G.; Tinaquero, D.; de Andrés, R.; Crespo, T.; Ponce-Balbuena, D.; Willis, B.C.; et al. Brugada Syndrome Trafficking-Defective Nav1.5 Channels Can Trap Cardiac Kir2.1/2.2 Channels. *JCI Insight* **2018**, *3*, 96291, doi:10.1172/jci.insight.96291.
6. Dago, M.; Crespo-García, T.; Cámara-Checa, A.; Rapún, J.; Rubio-Alarcón, M.; Marín, M.; Tamargo, J.; Caballero, R.; Delpón, E. Empagliflozin and Dapagliflozin Increase Na+ and Inward Rectifier K+ Current Densities in Human Cardiomyocytes Derived from Induced Pluripotent Stem Cells (hiPSC-CMs). *Cells* **2022**, *11*, 3707, doi:10.3390/cells11233707.
7. Crespo-García, T.; Rubio-Alarcón, M.; Cámara-Checa, A.; Dago, M.; Rapún, J.; Nieto-Marín, P.; Marín, M.; Cebrián, J.; Tamargo, J.; Delpón, E.; et al. A Cantú Syndrome Mutation Produces Dual Effects on KATP Channels by Disrupting Ankyrin B Regulation. *J Gen Physiol* **2023**, *155*, e202112995, doi:10.1085/jgp.202112995.
8. Waterhouse, A.; Bertoni, M.; Bienert, S.; Studer, G.; Tauriello, G.; Gumienny, R.; Heer, F.T.; de Beer, T.A.P.; Rempfer, C.; Bordoli, L.; et al. SWISS-MODEL: Homology Modelling of Protein Structures and Complexes. *Nucleic Acids Research* **2018**, *46*, W296–W303, doi:10.1093/nar/gky427.
9. Jiang, D.; Shi, H.; Tonggu, L.; Gamal El-Din, T.M.; Lenaeus, M.J.; Zhao, Y.; Yoshioka, C.; Zheng, N.; Catterall, W.A. Structure of the Cardiac Sodium Channel. *Cell* **2020**, *180*, 122-134.e10, doi:10.1016/j.cell.2019.11.041.
10. Akai, J.; Makita, N.; Sakurada, H.; Shirai, N.; Ueda, K.; Kitabatake, A.; Nakazawa, K.; Kimura, A.; Hiraoka, M. A Novel SCN5A Mutation Associated with Idiopathic Ventricular Fibrillation without Typical ECG Findings of Brugada Syndrome. *FEBS Lett* **2000**, *479*, 29–34, doi:10.1016/s0014-5793(00)01875-5.
11. Wei, J.; Wang, D.W.; Alings, M.; Fish, F.; Wathen, M.; Roden, D.M.; George, A.L. Congenital Long-QT Syndrome Caused by a Novel Mutation in a Conserved Acidic Domain of the Cardiac Na+ Channel. *Circulation* **1999**, *99*, 3165–3171, doi:10.1161/01.cir.99.24.3165.
12. Rajasekaran, N.; Suresh, S.; Gopi, S.; Raman, K.; Naganathan, A.N. A General Mechanism for the Propagation of Mutational Effects in Proteins. *Biochemistry* **2017**, *56*, 294–305, doi:10.1021/acs.biochem.6b00798.
